# Association between DCP levels and kidney stone prevalence in US female adults based on NHANES data

**DOI:** 10.1038/s41598-024-56832-6

**Published:** 2024-03-18

**Authors:** Heqian Liu, Jiawei Wang, Lingsong Tao, Yunwu Hao

**Affiliations:** 1 Department of Urology, The Second People’s Hospital of Wuhu City (Affiliated Wuhu Hospital of East China Normal University), Wuhu, 241000 China; 2 Department of Urology, Lu’an Hospital Affiliated of Anhui Medical University, Lu’an, 237000 Anhui China

**Keywords:** Kidney stones, 2,4-DCP and 2,5-DCP, Cross-sectional study, Environmental exposure, Environmental sciences, Urology

## Abstract

Our study aimed to evaluate the correlation between levels of 2,4-DCP(2,4-Dichlorophenol) and 2,5-DCP(2,5-Dichlorophenol) and the prevalence of kidney stones in US female adults. Participants were chosen from the National Health and Nutrition Examination Survey database, spanning the years 2007–2016. Dose–response curves were analyzed using logistic regression, subgroup analyses, and other statistical methods to evaluate the relationship between 2,4-DCP and 2,5-DCP levels and the prevalence of kidney stones. The final study included 3220 participants aged over 20 years, with 252 females reporting a history of kidney stones. After accounting for all interfering variables, we found that every 0.1 ug/ml increase in 2.4-DCP correlated with a 1% rise in kidney stone prevalence (OR = 1.01, 95% CI 1.00, 1.01), whereas the same increase in 2.5-DCP was linked to a 27% growth in prevalence (OR = 1.27, 95% CI 1.01, 1.61). Sensitivity analysis was performed by triangulating 2,4-DCP and 2,5-DCP levels. The dose–response curves demonstrated a linear positive relationship between 2,4-DCP and 2,5-DCP levels and the risk of stone development. Our findings indicate a positive correlation between 2,4-DCP and 2,5-DCP levels and the prevalence of kidney stones in US female adults. This association is of clinical significance; however, a direct causal relationship cannot be definitively established.

## Introduction

Kidney stones, among the most prevalent diseases of the urinary system, inflict significant physiological burdens on individuals. These burdens can lead to severe complications such as hydronephrosis, impairment of renal function, and, ultimately, renal insufficiency^1^. Beyond the physiological challenges, kidney stones also generate substantial economic costs, impacting both individuals and society at large. With a global prevalence ranging from 1 to 13%, kidney stones affect a significant proportion of the population, and the incidence is anticipated to rise further in the coming decades^2^.

The mechanism of stone formation is not merely a simple physicochemical disorder; it encompasses a series of complex processes, including crystal nucleation, growth, aggregation, and retention^3,4^. In exploring the formation of kidney stones, evidence suggests that, beyond the well-recognized genetic factors, stone development may be influenced by other elements such as climate, diet, exercise, and overall living environment^5,6^.

In particular, as society has developed, our living environments have undergone dramatic changes, and we are continually exposed to a vast array of chemical compounds in our daily lives and occupations. Literature reports indicate that environmental and pesticide exposure could be significant contributing factors to the increased prevalence of various diseases and metabolic syndromes, including obesity, neoplasms, neurological disorders, and type II diabetes mellitus, among others^7–10^.

Dichlorophenols (DCPs) are chemically synthesized endocrine disruptors that can be absorbed through the respiratory and gastrointestinal tracts and are primarily excreted in the urine^11–13^. Specific compounds such as 2,4-Dichlorophenol (2,4-DCP) and 2,5-Dichlorophenol (2,5-DCP), along with their precursors, are extensively employed in industrial and consumer products. Urinary DCPs serve as bio-detectors of human exposure to environmental chlorophenols and dichlorobenzene pesticides.

2,4-DCP is used mainly in the production of phenoxyacid herbicides, such as 2,4-diphenoxyacetic acid (2,4-D), as well as in the synthesis of pharmaceuticals and preservatives^14,15^. 2,5-DCP is a precursor of 1,4-diphenoxyacetic acid and is significant in the production of 1,4-dichlorophenol; it is the major metabolite of 1,4-dichlorobenzene (1,4-D) and is used in scenarios such as chemical intermediation in the manufacture of dyes, pharmaceuticals, and agricultural products, as well as an insect repellent and a space deodorizer for industrial and indoor household applications^16–18^.

Both 2,4-DCP and 2,5-DCP are also by-products of the chlorination of municipal drinking water and industrial wastewater, leading to frequent human exposure through gastrointestinal ingestion, dermal contact, and the utilization of products containing these chemicals^19,20^. From 2003 to 2010, 2,4-DCP and 2,5-DCP were detected in 81% of urine samples in the National Health and Nutrition Examination Survey (NHANES), a nationally representative survey, highlighting the ubiquity of these compounds in the environment.

Given the widespread exposure to these chemicals and animal studies indicating metabolic effects^21,22^, we assert that a comprehensive assessment of the association between these chemicals and common medical conditions is essential. We tested the hypothesis that elevated urinary concentrations of 2,4-DCP and 2,5-DCP correlate with the incidence of kidney stones by analyzing data from adult NHANES participants from 2007 to 2016. This is the pioneering study to propose that increased levels of 2,4-DCP and 2,5-DCP may be independently linked to the risk of kidney stones.

## Materials and methods

### Research population

The foundational clinical information for this study was obtained from the National Health and Nutrition Examination Survey (NHANES), spanning the years 2007–2016. Conducted as a cross-sectional survey, the U.S. Centers for Disease Control and Prevention (CDC) administers this survey biennially to a representative sample of the U.S. population.

As part of this national effort, NHANES orchestrates a series of physical examinations, laboratory tests, and questionnaires to garner information using probabilistic sampling methods. The Institutional Review Board of the National Center for Health Statistics (NCHS) carefully examined and approved the NHANES study protocol, and participants gave their consent by signing forms as part of the survey process.

**Figure 1:**
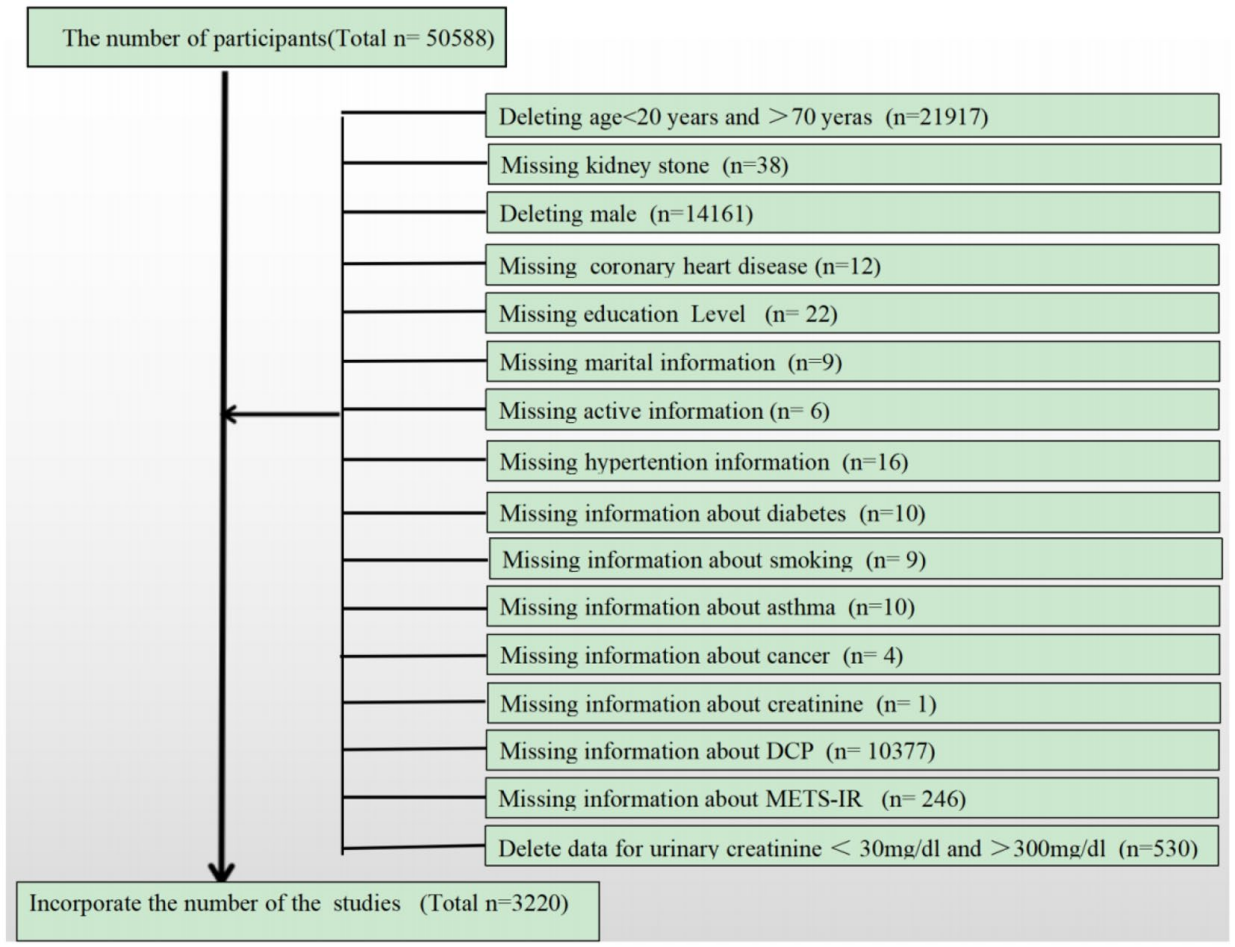
The participants selecting flow chart.

In our study, we assessed five successive 2-year survey cycles, particularly incorporating the Kidney Stone Questionnaire. We retained information from participants who explicitly responded to queries about their kidney stone history, resulting in a total of 50,588 participants involved in the questionnaire. The kidney stone questionnaire was only administered to those over the age of 20, so we removed participants under the age of 20. Data on DCPs in NHANES were collected in the subfile “Chemicals and Metabolites in Personal Care and Consumer Products,” and we believe that women between the ages of 20–70 years may be exposed to cosmetics more than other populations, and therefore our study included women between 20–70 years of age. We believe that women between the ages of 20–70 may be more likely to be exposed to cosmetics than the rest of the population, which may result in greater exposure to DCPs, and therefore we included women between the ages of 20–70 in our study. Figure 1 details the exclusion criteria for the study. Following the application of these criteria, the analysis included a final count of 3220 cases, with 252 females of them having a self-reported history of kidney stones.

### Data collection and definition

One-third of the NHANES participants were randomly chosen for DCP metabolite measurements. Two DCPs were detected in each of the five cycles of NHANES data. To mitigate bias resulting from readings below the limit of detection (LOD), only metabolites found in at least 75% of the samples were included. Every sample was examined spectroscopically through solid-phase extraction, in conjunction with high-performance liquid chromatography (HPLC) and tandem mass spectrometry (MS). Detailed information on these procedures is found in the Centers for Disease Control and Prevention (CDC) Laboratory Procedures Manual: DCP Metabolites.

For DCP sample concentrations below the detection level, the LOD was divided by the square root of two. Additionally, urine samples considered “unlikely to be normal” were removed—specifically, those too dilute (urine creatinine concentration < 30 mg/dL) or too concentrated (creatinine concentration > 300 mg/dL)^23^. Reference was made to prior studies where urinary creatinine was analyzed as an independent variable for diluting urine samples in the field^23,24^.

Kidney stones were assessed through questionnaires, with questions including a response to “Ever been told you have kidney stone?” to gauge self-reported status. An affirmative answer classified the participant as having kidney stones. The presence of kidney stones was treated as an outcome variable in the study.

Potential covariates that might confound the association between DCP and kidney stones were identified and included in multivariate-adjusted models. These covariates encompassed poverty-to-income ratio (PIR), age (in years), race, education level, alcohol consumption, marital status, physical activity, fasting cholesterol level (in mg/dl), serum creatinine (in mg/dl), urinary creatinine (mg/dl), smoking status (either smoker or non-smoker), and the presence of certain diseases such as hypertension, diabetes mellitus, asthma, coronary heart disease, and cancer. To further illustrate the impact of DCP on kidney stones, considering the metabolic syndrome and its effects on kidney stones and urinary phenols^25,26^, we additionally incorporated the insulin resistance index METS-IR into our analysis.

In our final analysis, we also considered dietary intake factors that may affect kidney stones, including energy, fat, and water intake. Every participant throughout the years underwent two 24-h dietary recalls, and the mean consumption derived from both recalls was utilized in our analyses.

In terms of handling missing values, when numerical variables displayed a significant number of missing entries, we transformed them into categorical variables. Specifically, we assessed these variables by transforming them into dichotomies at the median, using the lowest quartile as the reference group. The specific procedures used to measure the study's variables can be found publicly on the website www.cdc.gov/nchs/nhanes/.

### Statistical methods

In all statistical analyses, the NHANES study utilized sampling weights, stratification, and clustering to reflect the intricate, multi-stage sampling design. This approach ensured an accurate representation of the non-institutionalized U.S. population, providing accurate estimates without overstating statistical significance.

According to NHANES analysis guidelines, environmental samples were tested in one-third of the participants, and unique sub-weights were provided. New weights for the combined survey cycle were formulated by dividing the two-year weights for each individual cycle by two. The weights in the dataset were managed with the survey design R package in R programming language, following the guidelines on the official website.

To assess between-group differences, survey-weighted linear regression was used for continuous variables, while survey-weighted chi-square tests were applied to categorical variables. Continuous variables were represented as survey-weighted means along with 95% confidence intervals (CIs), and categorical variables were conveyed in a similar manner.

Covariate screening followed the STROBE guideline. A covariate was considered effective if its introduction into the base model or exclusion from the full model impacted the DCP regression coefficients by more than 10% and the *p* value for the covariate on the renal calculi regression coefficients was less than 0.1.

Multivariate logistic regression models were then utilized to explore the independent association between DCP and kidney stones in three distinct models:

- Model 1: Unadjusted.
- Model 2: Adjusted for age, race, education level, and marital status.
- Model 3: Adjusted for all variables except covariates with a variance inflation factor (VIF) greater than 5.

The relationship between each DCP and kidney stone was assessed separately. To delve deeper into the relationship, smoothed curve fitting was conducted using the penalized spline method, along with generalized additive model (GAM) regression.

Further multiple regression analyses were broken down by factors such as age, race, hypertension, and diabetes. A p-value below 0.05 was deemed statistically significant.

Empower software and R version 4.0.2 were utilized to carry out all the analyses.

### Informed consent

Informed consent was obtained from all subjects involved in the study.

### Institutional review board statement

The NCHS Research Ethics Review Committee approved the NHANES survey protocol (https://www.cdc.gov/nchs/nhanes/irba98.htm), and all participants of the study provided informed written consent. The NHANES database is open to the public and therefore the ethical review of this study was exempt. The study was conducted in accordance with the Declaration of Helsinki and was approved by the Institutional Review Board of the National Centre for Health Statistics.

## Result

### Higher levels of DCP found in the kidney stone group

The demographic characteristics of the 3220 participants (including 252 females, representing 7.82%) are detailed in Table 1. Among these individuals, those with kidney stones were found to have significantly higher levels of 2,4-DCP and 2,5-DCP. Specifically, the renal stone patients had levels of 4.52 (0.23, 8.81) ng/ml for 2,4-DCP and 138.77 (75.21, 282.75) ng/ml for 2,5-DCP, compared to 3.63 (2.84, 4.42) ng/ml and 111.21 (79.16, 143.26) ng/ml, respectively, in those without renal stones.

**Table 1:** Baselines characteristics of participants, weighted.

Characteristic	Nonstone formers	Stone formers	*P* value
	N = 2968	N = 252	
Age (years)	43.32 (42.59, 44.05)	45.23 (42.45, 48.01)	0.1918
Serum cholesterol (mg/dl)	196.90 (194.71, 199.08)	195.99 (189.86, 202.12)	0.7752
Serum calcium (mg/dl)	9.36 (9.34, 9.38)	9.37 (9.31, 9.43)	0.8472
Serum creatinine (mg/dl)	0.75 (0.74, 0.76)	0.76 (0.72, 0.79)	0.6086
Urine creatinine (mg/dl)	112.11 (108.81, 115.42)	109.25 (102.20, 116.30)	0.4758
METS-IR index	2.27 (2.26, 2.29)	2.33 (2.28, 2.38)	0.0466
2,5-Dichlorophenol (ng/ml)	111.21 (79.16, 143.26)	138.77 (75.21, 282.75)	0.043
2,4-Dichlorophenol (ng/ml)	3.63 (2.84, 4.42)	4.52 (0.23, 8.81)	0.024
*Race (%)*			0.0537
Mexican American	15.64 (13.16, 18.50)	15.96 (10.80, 22.96)	
White	62.87 (59.10, 66.49)	70.84 (61.96, 78.36)	
Black	13.54 (11.52, 15.85)	7.09 (4.63, 10.72)	
Other race	7.94 (6.71, 9.38)	6.11 (2.93, 12.30)	
*Education level (%)*			0.9987
Less than high school	14.87 (13.04, 16.90)	14.77 (10.45, 20.46)	
High school	20.90 (18.79, 23.17)	20.81 (14.60, 28.77)	
More than high school	64.24 (60.79, 67.54)	64.42 (55.31, 72.60)	
*Marital status (%)*			0.8364
Cohabitation	63.14 (60.15, 66.04)	63.95 (55.74, 71.41)	
Solitude	36.86 (33.96, 39.85)	36.05 (28.59, 44.26)	
*Alcohol (%)*			0.6226
Yes	65.08 (62.21, 67.84)	68.66 (61.04, 75.40)	
No	25.55 (23.08, 28.19)	22.95 (17.22, 29.90)	
Unclear	9.37 (7.91, 11.08)	8.39 (5.23, 13.18)	
*High blood pressure (%)*			< 0.0001
Yes	25.97 (24.07, 27.97)	44.98 (36.20, 54.09)	
No	74.03 (72.03, 75.93)	55.02 (45.91, 63.80)	
*Diabetes (%)*			0.0001
Yes	7.53 (6.35, 8.92)	16.79 (11.26, 24.30)	
No	92.47 (91.08, 93.65)	83.21 (75.70, 88.74)	
*Smoked (%)*			0.0054
Yes	36.82 (34.19, 39.54)	49.26 (40.23, 58.33)	
No	63.18 (60.46, 65.81)	50.74 (41.67, 59.77)	
*Physical activity (%)*			0.0081
Never	29.12 (26.75, 31.61)	37.97 (30.14, 46.48)	
Moderate	38.43 (36.27, 40.64)	40.99 (33.66, 48.75)	
Vigorous	32.45 (30.23, 34.76)	21.03 (15.61, 27.72)	
*Asthma (%)*			0.2284
Yes	83.46 (81.54, 85.20)	79.67 (72.28, 85.49)	
No	16.54 (14.80, 18.46)	20.33 (14.51, 27.72)	
*Coronary artery disease (%)*			0.8301
Yes	1.51 (1.03, 2.20)	1.32 (0.41, 4.21)	
No	98.49 (97.80, 98.97)	98.68 (95.79, 99.59)	
*Cancers (%)*			0.7355
Yes	8.58 (7.22, 10.16)	7.72 (4.16, 13.88)	
No	91.42 (89.84, 92.78)	92.28 (86.12, 95.84)	
*PIR (%)*			0.7381
< 1.3	22.81 (20.93, 24.81)	25.28 (19.14, 32.59)	
≥ 1.3 < 3.5	31.20 (28.98, 33.51)	27.20 (19.59, 36.43)	
≥ 3.5	39.24 (36.76, 41.77)	41.49 (32.45, 51.14)	
Unclear	6.76 (5.61, 8.12)	6.03 (3.04, 11.58)	
*Total Kcal (%)*			0.1299
Lower	41.15 (38.89, 43.45)	33.73 (25.51, 43.07)	
Higher	44.69 (42.07, 47.33)	46.89 (38.14, 55.84)	
Unclear	14.16 (12.58, 15.91)	19.38 (13.69, 26.70)	
*Total sugar (%)*			0.5898
Lower	37.80 (35.44, 40.21)	38.44 (30.72, 46.78)	
Higher	36.13 (33.73, 38.59)	32.32 (24.85, 40.83)	
Unclear	26.08 (24.11, 28.14)	29.24 (22.11, 37.56)	
*Total water (%)*			0.2377
Lower	39.78 (37.50, 42.09)	36.57 (28.04, 46.04)	
Higher	46.06 (43.47, 48.67)	44.05 (36.12, 52.30)	
Unclear	14.16 (12.58, 15.91)	19.38 (13.69, 26.70)	
*Total fat (%)*			0.2377
Lower	39.78 (37.50, 42.09)	36.57 (28.04, 46.04)	
Higher	46.06 (43.47, 48.67)	44.05 (36.12, 52.30)	
Unclear	14.16 (12.58, 15.91)	19.38 (13.69, 26.70)	

For continuous variables: survey-weighted mean (95% CI), *P* value was by survey-weighted linear regression (svyglm).

For categorical variables: survey-weighted percentage (95% CI), *P* value was by survey-weighted Chi-square test (svytable).

### Association between higher DCP levels and increased prevalence of kidney stones

In the final statistical model, all covariates that had VIF values under 5 were included. The logistic regression results revealed a significant association between DCP exposure and kidney stones. For ease of interpretation, we scaled down the DCP levels by an average of 100-fold, resulting in final units of 0.1 ug/ml.

In the fully adjusted model 3:

A 0.1 ug/ml increase in 2,4-DCP was associated with a 1% increase in kidney stone prevalence (OR = 1.01, 95% CI 1.00, 1.01).

A 0.1 ug/ml rise in 2,5-DCP was associated with a 27% growth in the occurrence of kidney stones (OR = 1.27, 95% CI 1.01, 1.61).

**Figure 2:**
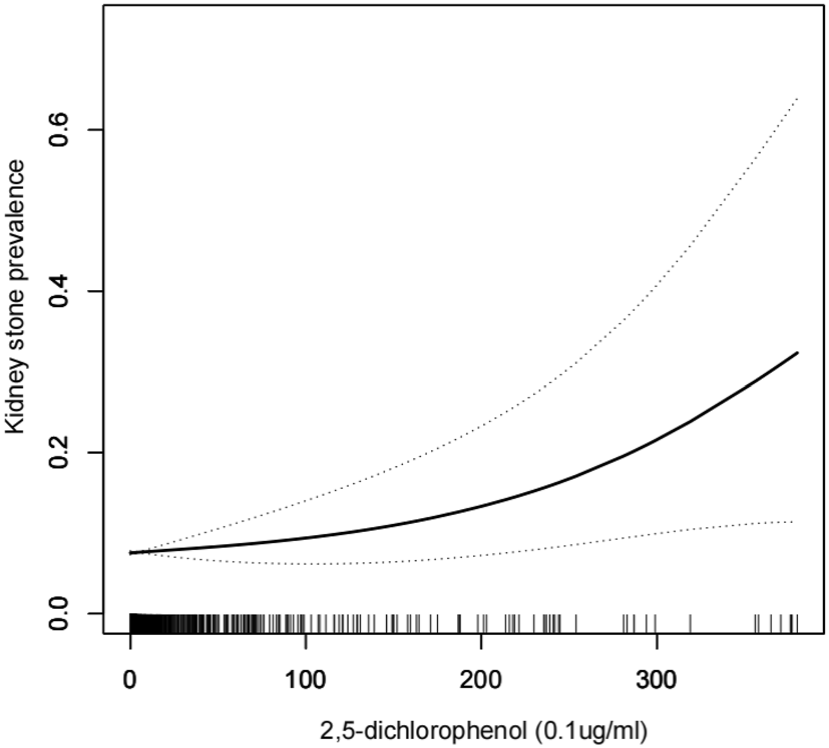
Density dose–response relationship between 2,5-DCP with kidney stone prevalence. The area between the upper and lower dashed lines is represented as 95% CI. Each point shows the magnitude of the index and is connected to form a continuous line.Adjusted for all covariates except effect modifier.

**Figure 3:**
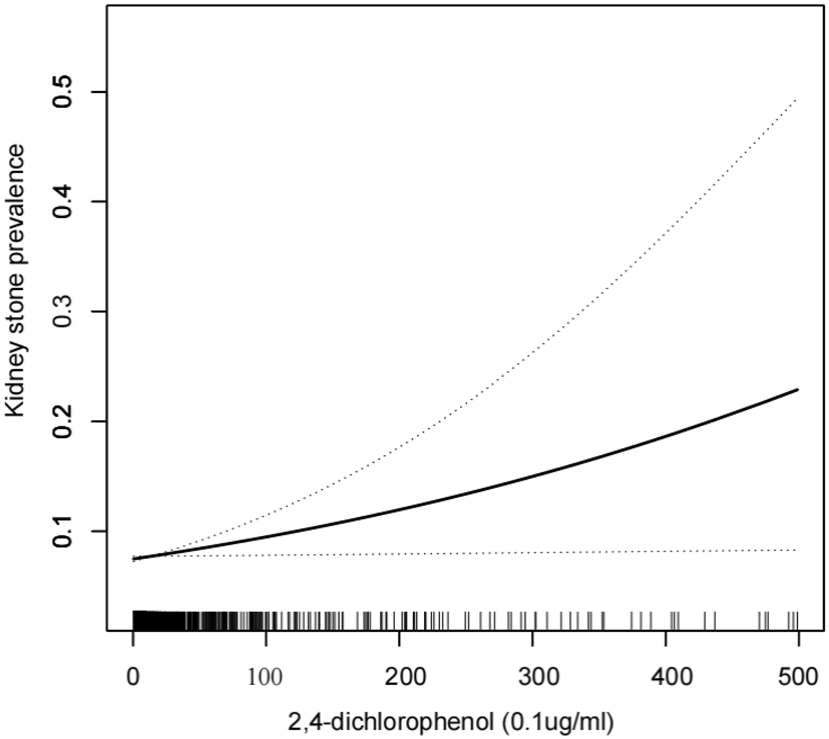
**A** Density dose–response relationship between 2,4-DCP with kidney stone prevalence. The area between the upper and lower dashed lines is represented as 95% CI. Each point shows the magnitude of the index and is connected to form a continuous line. Adjusted for all covariates except effect modifier.

Smoothed curve fitting revealed a linear positive correlation between both DCP levels and kidney stone prevalence (Table 2, Figs. 2, 3).

**Table 2:** Logistic regression analysis between DCP with kidney stone prevalence.

Characteristic	Model 1 OR (95% CI)	Model 2 OR (95% CI)	Model 3 OR (95% CI)
2,5-Dichlorophenol (0.1ug/ml)	1.01 (1.00, 1.01)	1.01 (1.00, 1.01)	**1.01 (1.00, 1.01)**
*Stratified by age*			
20–39	0.95 (0.87, 1.05)	0.97 (0.90, 1.06)	0.98 (0.90, 1.06)
40–70	1.01 (1.00, 1.02)	1.01 (1.00, 1.02)	**1.01 (1.00, 1.02)**
*Stratified by race*			
Mexican American	1.00 (0.98, 1.02)	1.00 (0.98, 1.02)	1.00 (0.98, 1.03)
White	1.01 (0.96, 1.06)	1.01 (0.96, 1.06)	1.01 (0.97, 1.07)
Black	1.01 (1.00, 1.02)	1.01 (1.00, 1.02)	**1.01 (1.00, 1.02)**
Other race	1.00 (0.89, 1.11)	0.98 (0.87, 1.12)	0.97 (0.84, 1.11)
2,4-Dichlorophenol (0.1ug/ml)	1.21 (0.96, 1.52)	1.28 (1.01, 1.61)	**1.27 (1.01, 1.61)**
*Stratified by age*			
20–39	0.06 (0.00, 5.71)	0.13 (0.00, 9.69)	0.13 (0.00, 11.64)
40–70	1.26 (0.98, 1.61)	1.31 (1.03, 1.68)	**1.33 (1.03, 1.70)**
*Stratified by race*			
Mexican American	0.82 (0.28, 2.42)	0.79 (0.23, 2.66)	0.84 (0.25, 2.87)
White	0.88 (0.09, 8.80)	0.83 (0.08, 8.96)	0.88 (0.05, 14.17)
Black	1.36 (1.04, 1.77)	1.35 (1.04, 1.75)	**1.35 (1.01, 1.80)**
Other Race	1.07 (0.03, 44.28)	0.68 (0.01, 64.86)	0.27 (0.00, 52.93)

Model 1 was adjusted for no covariates;

Model 2 was adjusted for age, race, marital status and education;

Model 3 was adjusted for adjusted for all covariates except effect modifier.

Bold text indicates the result is significant in model3.

### Subgroup analysis

We conducted a stratified analysis based on age and race, finding consistent results across the middle two DCP groups. For 2,4-DCP:

A rise in kidney stone prevalence was observed in individuals over 40 years of age (OR = 1.01, 95% CI: 1.00, 1.02) and within the black racial group (OR = 1.01, 95% CI 1.00, 1.02).

For 2,5-DCP:

An elevated level was associated with increased kidney stone prevalence in individuals aged > 40 years (OR = 1.33, 95% CI 1.03, 1.70) and in the black racial group (OR = 1.35, 95% CI 1.01, 1.80).

These results indicate that elevated levels of 2,4-DCP and 2,5-DCP could be associated with a heightened risk of developing kidney stones.

## Discussion

Environmental and pesticide exposures are known to correlate with numerous human health outcomes, and they represent a critical issue in both global public health and environmental stewardship. However, there is a lack of research regarding the association of 2,4-DCP and 2,5-DCP levels with kidney stones.

In our analysis of kidney stone patients with exposure to 2,4-DCP and 2,5-DCP in the NHANES database from 2007 to 2016, we found higher levels of DCP in the kidney stone group. This observation indicates that higher DCP levels might be linked to a rise in the occurrence of kidney stones. Furthermore, we conducted a sex- and ethnicity-stratified analysis and determined that individuals over the age of 40 (OR = 1.01, 95% CI 1.00, 1.02) and those with elevated 2.4-DCP levels within the black group (OR = 1.01, 95% CI 1.00, 1.02) were more likely to have kidney stones. Similar findings were noted for elevated 2.5-DCP levels in the age group over 40 years (OR = 1.33, 95% CI 1.03, 1.70) and the black group (OR = 1.35, 95% CI 1.01, 1.80). These results reinforce the potential value of DCP as an indicator for kidney stone development and prediction.

Additionally, a recent study indicated that urinary levels of 2,4-DCP and 2,5-DCP were linked to several common diseases. For instance, higher urinary concentrations of 2,5-DCP were correlated with an increased prevalence of heart disease and all combined cancers, although no significant associations were found with lung, thyroid, or liver diseases^27^. Regarding diabetes, a dose-dependent relationship was identified between urinary 2,5-DCP and a higher prevalence of the condition, while 2,4-DCP was not significantly linked to diabetes prevalence. Moreover, elevated 2,5-DCP concentrations have been related to increased thyroid-stimulating hormone levels^28^, which could contribute to hypothyroidism and subsequent weight gain^29^. In a study focusing on children's environmental health, 2,5-DCP was positively associated with the prevalence of asthma and related symptoms in boys but not in girls^30^.

Regarding the specific mechanism of action of DCP, some scholars have conducted in vitro studies and constructed animal models. In these in vitro studies, chlorinated phenols were found to be prone to uncoupling oxidative phosphorylation, possibly leading to intracellular ATP depletion. Additionally, p-DCB demonstrated estrogenic activity in both in vitro and in vivo animal studies^22^. Chronic exposure to p-DCB has been linked to liver and kidney damage^31^, and both high-dose (300 mg/kg/day) and low-dose (150 mg/kg/day) administration of p-DCB were shown to increase cell proliferation in the livers of rats and mice^32^.

Based on our findings, we can confirm a significant correlation between 2,4-DCP and 2,5-DCP levels and the prevalence of kidney stones. To identify specific populations at risk and to improve kidney stone prevention, we performed a subgroup analysis. In the age subgroup analysis, the correlation between elevated 2,4-DCP and 2,5-DCP levels and kidney stone prevalence was stronger in the > 40 age group compared to the < 40 group. This result is encouraging, suggesting that testing 2,4-DCP and 2,5-DCP levels in individuals over 40 may be a more effective strategy for preventing kidney stone development in middle-aged and older adults.

As per the U.S. National Health and Nutrition Examination Survey (NHANES), conducted between 2003 and 2010, a level of 0.88 μg/L of 2,4-DCP was noted in individuals aged 20–59 years^21^. In a parallel manner, a German study documented a level of 0.54 μg/L in those aged 18–69 years^33^. Since 2,4-DCP levels don't seem to change notably with age, our results have implications for both the prevention and management of kidney stones in individuals over the age of 40.

Regarding ethnic subgroups, 2,4-DCP and 2,5-DCP levels were more strongly associated with kidney stone prevalence in the black group compared to the white group. Other NHANES studies have provided evidence that non-Hispanic whites tend to have significantly lower concentrations of DCP compared to Hispanic-dental or non-Hispanic blacks^21,34^. Consequently, racial differences may contribute to variations in urinary dichlorophenol concentrations, reinforcing the reasonableness of our conclusions.

Our study boasts several strengths. First, NHANES rigorously adhered to a well-designed study protocol, providing a wealth of high-quality data that includes measurements of many environmental pollutants of potential public health significance. The consistency of the data, even when considering sample weighting issues, adds to the credibility of the findings. Furthermore, the results are broadly applicable to the general population of the U.S., thanks to a large sample size that allowed for relevant subgroup analyses, affirming the robustness of the results.

Despite these strengths, our study is not without limitations:

The cross-sectional design of the study means that we were unable to determine a causal relationship between 2,4-DCP and 2,5-DCP levels and the prevalence of renal stones.

Although we adjusted for potential covariates, there is still a possibility that the results could be affected by the presence of unknown variables, such as urinary calcium, urinary pH, and use of fast-food consumption, which can adversely affect the results, but these data are not yet available in NHANES, and we therefore hope that more data will be disclosed in the future.

The information about renal stones was obtained from questionnaires, so recall bias is unavoidable. It's also unclear whether the one-time measurements accurately reflect long-term exposure to DCP. Additionally, the presence of asymptomatic renal stones might have adversely affected our study, creating potential biases in our findings.

## Conclusion

This study indicates that increased levels of 2,4-DCP and 2,5-DCP are correlated with a greater prevalence of kidney stones. Evaluating 2,4-DCP and 2,5-DCP levels could prove beneficial to kidney health, particularly for middle-aged and older adults who may stand to benefit more from these assessments. However, additional research is required to confirm our findings.

## Data Availability

The data used or analyzed in this study are available from the corresponding author upon reasonable request.
